# The impact of immobilisation and inflammation on the regulation of muscle mass and insulin resistance: different routes to similar end‐points

**DOI:** 10.1113/JP275444

**Published:** 2018-08-18

**Authors:** Hannah Crossland, Sarah Skirrow, Zudin A. Puthucheary, Dumitru Constantin‐Teodosiu, Paul L. Greenhaff

**Affiliations:** ^1^ MRC/Arthritis Research UK Centre for Musculoskeletal Ageing Research, Arthritis Research UK Centre for Sport, Exercise and Osteoarthritis, National Institute for Health Research Nottingham Biomedical Research Centre, School of Life Sciences University of Nottingham UK; ^2^ Institute of Sport, Exercise and Health London UK; ^3^ Royal Free NHS Foundation Trust London UK

**Keywords:** carbohydrate metabolism, muscle fuel selection, inactivity, bed-rest, muscle atrophy, muscle protein synthesis, muscle protein breakdown

## Abstract

Loss of muscle mass and insulin sensitivity are common phenotypic traits of immobilisation and increased inflammatory burden. The suppression of muscle protein synthesis is the primary driver of muscle mass loss in human immobilisation, and includes blunting of post‐prandial increases in muscle protein synthesis. However, the mechanistic drivers of this suppression are unresolved. Immobilisation also induces limb insulin resistance in humans, which appears to be attributable to the reduction in muscle contraction *per se*. Again mechanistic insight is missing such that we do not know how muscle senses its “inactivity status” or whether the proposed drivers of muscle insulin resistance are simply arising as a consequence of immobilisation. A heightened inflammatory state is associated with major and rapid changes in muscle protein turnover and mass, and dampened insulin‐stimulated glucose disposal and oxidation in both rodents and humans. A limited amount of research has attempted to elucidate molecular regulators of muscle mass loss and insulin resistance during increased inflammatory burden, but rarely concurrently. Nevertheless, there is evidence that Akt (protein kinase B) signalling and FOXO transcription factors form part of a common signalling pathway in this scenario, such that molecular cross‐talk between atrophy and insulin signalling during heightened inflammation is believed to be possible. To conclude, whilst muscle mass loss and insulin resistance are common end‐points of immobilisation and increased inflammatory burden, a lack of understanding of the mechanisms responsible for these traits exists such that a substantial gap in understanding of the pathophysiology in humans endures.

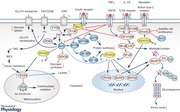

## Introduction

Loss of muscle mass and insulin sensitivity are common phenotypic traits of immobilisation (e.g. bed‐rest or limb casting) as well as being associated with ageing, inflammation and trauma, and chronic non‐communicable disease. In particular, sepsis is associated with major metabolic alterations, including significant losses of muscle mass (Hasselgren *et al*. 2005) and hyperglycaemia (Mizock, 2001), dysregulation of fat and carbohydrate utilisation (Saeed *et al*. 1999; Chambrier *et al*. 2000) and hyperlactataemia (Vary, 1999) that is consistent with the impairment of muscle carbohydrate oxidation and insulin resistance. However, our mechanistic understanding of the aetiology of such metabolic perturbations in immobilisation, acute trauma, sepsis and chronic non‐communicable disease is currently poor, particularly in humans, as is detailed insight of whether these negative traits can be at least partly rescued by interventions such as exercise and/or targeted drug administration in humans. Clarity of understanding towards these gaps in our understanding and new insight regarding the trajectories of change and the molecular drivers of muscle mass loss and insulin resistance is vital if in‐roads are to be made in preserving muscle mass and metabolic health in these scenarios. We therefore focus attention here on the impact of immobilisation and inflammation on the loss of muscle mass and insulin sensitivity in humans.

## Immobilisation

### Immobilisation induced loss of muscle mass

The maintenance of muscle mass is dependent on the balance between rates of muscle protein synthesis and muscle protein breakdown, where a chronic imbalance results in either the loss or gain of muscle mass. *In vivo* animal studies utilising stable isotope tracer methodologies to quantify muscle protein turnover in an acute setting conclude that muscle protein synthesis is less (Booth & Seider, 1979) and muscle protein breakdown is greater (Kobayashi *et al*. 2006) following both 6 and 24 h of cast immobilisation compared to the basal non‐immobilised state. Furthermore, evidence from research involving administration of the proteasome inhibitor velcade to rodents during 3 days of limb immobilisation resulted in an ∼50% sparing of muscle weight, leading to the suggestion that muscle protein breakdown predominates in the rodent during immobilisation (Krawiec *et al*. 2005). In contrast to rodent studies, it is thought that suppression of muscle protein synthesis is the primary driver of muscle mass loss in the immobilised state in humans (Phillips *et al*. 2009; Murton & Greenhaff, 2010). For example, De Boer and colleagues detected a 50% decline in the rate of post‐absorptive myofibrillar protein synthesis measured over several hours following 10 days of limb suspension in healthy, young volunteers when compared to baseline. The authors concluded that the decline in myofibrillar protein synthesis, even in these fasted state conditions, was of sufficient magnitude to fully account for the decline in muscle cross‐sectional area recorded in the same volunteers, i.e. the contribution from muscle protein breakdown to total muscle mass loss was paltry (De Boer *et al*. 2007). Reasons for these inconsistencies probably reside in the undoubted metabolic differences between humans and rodents, most notably the relatively greater rates of muscle protein synthesis and basal metabolic rate in rodents that have limited capacity to maintain metabolic homeostasis (Demetrius, 2005). Furthermore, most rodent studies invariably involve young animals that are still very much in their growth phase compared to adult humans, whilst the magnitude of immobilisation induced stress is likely to be far greater for rodents than for consenting human volunteers. The influence these compounding issues may have is exemplified by the degree of muscle mass loss observed between species, where 3 days of hindlimb cast immobilisation of a rodent has been shown to result in an ∼19% lower muscle mass compared to time‐matched controls (Krawiec *et al*. 2005), *vs*. an ∼5% decline in human quadriceps mass after 2 weeks of full‐limb cast immobilisation (Jones *et al*. 2004). However, it is important to recognise that the body of evidence published to date does not preclude a role for muscle protein breakdown during disuse atrophy in humans. Indeed, increased amounts of ubiquitin protein conjugates (Glover *et al*. 2008) and increased 3‐methylhistidine (Tesch *et al*. 2008) release in the first few days of muscle disuse point to an early and possibly transient contribution of muscle protein breakdown to the aetiology of human disuse atrophy. However, whilst any contribution is likely to be comparatively small, the current lack of firm evidence based on the application of tracer methodologies over the time course of muscle immobilisation in humans makes the exact contribution of muscle protein breakdown to the aetiology of disuse‐induced muscle loss in humans speculative. Of note, recent evidence reports a 6‐fold increase in a small number of NCAM (also known as CD56; see Table 1 for definitions of abbreviations) positive muscle fibres following 3 days of immobilisation in healthy male volunteers, pointing to an early denervation process which would presumably ultimately involve changes in both muscle protein synthesis and degradation in these fibres (Demangel *et al*. 2017).

**Table 1:** Abbreviations

4E‐BP1	Eukaryotic translation initiation factor 4E binding protein 1
Akt	Serine/threonine‐specific protein kinase
CRP	C‐reactive protein
E2	E2 ubiquitin‐conjugating enzyme
eEF2	Eukaryotic elongation factor‐2
eIF‐4E	Eukaryotic translation initiation factor 4E
FAT/CD36	Fatty acid translocase/Cluster of differentiation 36
FOXO	Forkhead box O
GLUT4	Glucose transporter 4
GSK3α and β	Glycogen synthase kinase 3 α and β
IκB	Inhibitor of NF‐κB
IL‐1 and ‐6	Interleukin 1 and 6
IMCL	Intramyocellular lipid
IRS‐1	Insulin receptor signalling protein 1
MAFbx	Muscle atrophy F‐box
mTOR	Mammalian target of rapamycin
MuRF1	Muscle ring finger‐1
MYD88	Myeloid differentiation primary response protein
NCAM	Neural cell adhesion molecule
NCAM/CD56	Neural cell adhesion molecule/Cluster of differentiation 56
NF‐κB	Nuclear Factor Kappa Beta
p70S6K	Ribosomal protein S6 kinase β‐1
PDC	Pyruvate dehydrogenase complex
PDK	Pyruvate dehydrogenase kinase
Pi	Inorganic phosphate
PIP	phosphatidylinositol‐1,4,5‐trisphosphate
PI3K	Phosphoinositide 3‐kinase
PTEN	Phosphatase and tensin homolog
Smad2,3	Homologies to the *Caenorhabditis elegans* (SMA) and Drosophila (AD) family of genes for receptors of the transforming growth factor beta (TGF‐B) superfamily
TAG	Triglyceride
TLR4	Toll‐like receptor 4
TNFR1	Tumor necrosis factor receptor
TRIF	TIR‐domain‐containing adapter‐inducing interferon‐β
Ub	Ubiquitin
TNFα	Tumour necrosis factor α

### Cellular and molecular mechanisms allied to immobilisation induced muscle mass loss

Research has highlighted protein translation initiation, where the ribosomal structure is formed and the associated mRNA transcript becomes bound in response to increased intramuscular amino acid availability and/or muscle contraction, as a key point of regulation of muscle protein synthesis, including in a number of conditions where a decline in the rate of muscle protein synthesis is observed (Vary & Kimball, 1992, 2000; Vary *et al*. 1994). The Akt/mTOR/p70S6K signalling cascade has been assigned a central role in this nutrient and/or contraction mediated activation of protein translation initiation, and is founded on experiments demonstrating that high frequency electrical stimulation of rodent muscle occurs in parallel with increased phosphorylation of these signalling proteins (Atherton *et al*. 2005) and muscle specific over‐expression of Akt in transgenic mice results in muscle hypertrophy (Bodine *et al*. 2001). However, the established notion that increased phosphorylation of Akt/mTOR/p70S6K signalling proteins is a central regulator of muscle protein synthesis in human muscle is debatable given that a disassociation between signalling protein phosphorylation and muscle protein turnover has been demonstrated. For example, stepwise increases in serum insulin concentration at a known and fixed amino acid infusion rate failed to modulate leg protein synthesis any further than amino acid administration alone (with insulin maintained at the post‐absorptive concentration), despite markedly increasing muscle Akt and p70S6K phosphorylation (Greenhaff *et al*. 2008). Similarly, Wilkinson *et al*. (2008) demonstrated that acute resistance exercise, but not endurance exercise, was able to increase post‐exercise myofibrillar protein synthesis despite signalling protein phosphorylation being increased to the same extent in both exercise protocols. From the perspective of human immobilisation, evidence suggests that the Akt/mTOR/p70S6K signalling cascade has no obvious role in the decline in muscle protein synthesis given that neither the phosphorylation state nor content of Akt, p70S6K, 4E‐BP1 or eIF‐4E were altered in the post‐absorptive state following 10 or 21 days of limb suspension (de Boer *et al*. 2007). Furthermore, although immobilisation blunted the increase in muscle protein synthesis in response to amino acid infusion in healthy volunteers when compared to the non‐immobilised contralateral limb (even under conditions of high amino acid provision), this anabolic blunting occurred in the face of similar changes in the phosphorylation state of the Akt/mTOR/p70S6K signalling pathway in both limbs (Glover *et al*. 2008), highlighting that this pathway cannot be regulating the deficits in post‐absorptive or post‐prandial muscle protein synthesis observed during immobilisation. On balance, it would seem that the precise mechanisms responsible for the decline in muscle mass observed during immobilisation in humans are not at all clear, and available data cast doubt on the measure of protein phosphorylation being a robust proxy of Akt/mTOR/p70S6K signalling pathway flux.

### Immobilisation induced muscle insulin resistance

Bed‐rest induces whole body insulin resistance in human volunteers, suggested by the increase in post‐absorptive blood glucose and serum insulin concentrations, and more definitively by reductions in blood glucose clearance following an oral glucose challenge and whole body glucose clearance during an hyperinsulinaemic euglycaemic insulin clamp (Mikines *et al*. 1991; Sonne *et al*. 2010, 2011). These adaptations occur within just 3–5 days of immobilisation (Stuart *et al*. 1988; Smorawinski *et al*. 2000), and because they are also evident at a local limb level are interpreted as being representative of skeletal muscle insulin resistance. Additionally, insulin resistance develops after 3–14 days of reduced ambulatory activity (Olsen *et al*. 2008; Krogh‐Madsen *et al*. 2010). Collectively these studies suggest the reduction in muscle contraction *per se* drives the deficits in post‐prandial glucose disposal, which may also explain the association between age and insulin resistance reported in the literature. However, the mechanisms underpinning this physiological response are unclear and are considered further below.

### Cellular and molecular mechanisms associated with immobilisation induced muscle insulin resistance

Under normal physiological conditions, skeletal muscle glucose transport is a rate limiting step in blood glucose disposal and is thought to occur as a result of blunted insulin signalling and/or GLUT 4 translocation to the plasma membrane (Zierath *et al*. 1996; Garvey *et al*. 1998). Maintenance of insulin signalling via the IRS‐1/Akt pathway seems to be essential for insulin mediated muscle glucose uptake because of its involvement in GLUT4 translocation and its dysregulation in type 2 diabetes (Björnholm *et al*. 1997; Kim *et al*. 1999; Morino *et al*. 2005). Moreover in the context of this review, rodent hindlimb immobilisation has been shown to reduce IRS‐1 protein expression and Akt activity (Hirose *et al*. 2000), whilst bed‐rest has been shown to blunt insulin stimulated Akt phosphorylation in humans (Kiilerich *et al*. 2011; Mortensen *et al*. 2013). Furthermore, 7–19 days bed‐rest (Tabata *et al*. 1999; Op ‘t Eijnde *et al*. 2001; Biensø *et al*. 2012) has been shown to reduce muscle GLUT4 protein content. In keeping with a decline in insulin mediated glucose uptake, reduced muscle hexokinase activity and/or expression has also been observed following immobilisation (Ringholm *et al*. 2011; Biensø *et al*. 2012), alongside a decline in muscle glycogen synthase activity (Biensø *et al*. 2012). Whether any of these responses are drivers of immobilisation induced muscle insulin resistance or occur as a consequence of it is unknown. Indeed, we do not yet know how muscle senses its “inactivity status” or the true source of immobilisation induced muscle insulin resistance.

Another important candidate for immobilisation induced impairment of muscle glucose disposal is IMCL accumulation. A positive correlation exists between post‐absorptive plasma free fatty acid concentrations and whole body insulin resistance, and the relationship between IMCL content and insulin resistance is even stronger (Pan *et al*. 1997 *a*; Krssak *et al*. 1999). Furthermore, IMCL content is positively correlated with insulin resistance in healthy volunteers and in first degree relatives of patients with type 2 diabetes (Krssak *et al*. 1999; Perseghin *et al*. 1999). It seems logical therefore that IMCL content may play a central role in the aetiology of skeletal muscle insulin resistance during immobilisation. Indeed, an increase in IMCL content has been observed following 28 days of unilateral lower limb suspension and bed‐rest (Manini *et al*. 2007; Cree *et al*. 2010), which was less obvious after 7 days bed‐rest (Dirks *et al*. 2016). An increase in IMCL content during immobilisation could originate from excessive free fatty acid supply secondary to positive energy intake and/or reduced physical activity. Accordingly, the use of acipimox to reduce plasma free fatty acid concentration and IMCL content resulted in a marked improvement in insulin sensitivity in non‐immobilised volunteers (Bajaj *et al*. 2004). From a mechanistic standpoint, the accumulation of IMCL will increase intracellular fatty acid metabolites, such as long chain fatty acyl CoAs, long chain acylcarnitines, diacylglycerols and ceramides, which collectively will blunt insulin signalling and/or inhibit pyruvate dehydrogenase flux also reducing muscle glucose utilisation. What is the mechanism behind the immobilisation induced increase in IMCL? Physical inactivity reduces lipid oxidation (Ritz *et al*. 1998; Bergouignan *et al*. 2006), which has been attributed to a reduction in metabolic rate in this situation (Blanc *et al*. 2000). Accordingly, the decline in lipid oxidation observed after 7 days bed‐rest was paralleled by the development of whole body insulin resistance (Blanc *et al*. 2000). One interpretation of these data therefore is that an increase in IMCL content results directly from an immobilisation induced reduction in the rate of lipid oxidation in tandem with a reduction in muscle metabolic rate and a parallel increase in plasma free fatty acid availability. What the time course of muscle IMCL accumulation is during immobilisation and how this relates to the temporal change in muscle insulin sensitivity is unknown. Indeed, whether muscle IMCL accumulation is a driver of immobilisation induced muscle insulin resistance or a consequence of it is unknown.

Finally, a decrease in mitochondrial content and/or mitochondrial function during immobilisation has also been proposed as a potential driver of altered fuel metabolism under these conditions. In support of this, microarray analysis revealed altered mRNA expression of genes involved in mitochondrial bioenergetics and carbohydrate metabolism after 2 and 14 days of immobilisation in healthy volunteers (Abadi *et al*. 2009). Furthermore, 14 days of immobilisation reduced quadriceps muscle mitochondrial respiratory capacity and protein content, and to the same extent in young and old volunteers (Gram *et al*. 2014). Importantly, this response was shown to be a function of a decrease in mitochondrial content that accompanies immobilisation rather than a change in intrinsic mitochondrial function *per se*. This is based on the evidence that the immobilisation induced decline in mitochondrial respiratory capacity disappeared when respiration was normalised to citrate synthase activity (a marker of mitochondrial content). In keeping with this, exercise training following immobilisation restored mitochondrial capacity and citrate synthase activity (Gram *et al*. 2014). Nevertheless, the same group of authors were able to subsequently show increased mitochondrial reactive oxygen species production in the face of no change in anti‐oxidant capacity, following immobilisation, which was accompanied by decreased mitochondrial ATP generating respiration. However, the consequences of this with respect to altered fuel metabolism under these conditions were not investigated. Exercise training following immobilisation did, however, restore both (Gram *et al*. 2015).

## Inflammation

### Inflammation induced loss of muscle mass

Several pro‐inflammatory cytokines (e.g. CRP, IL‐1, IL‐6 and TNFα) have been repeatedly implicated in altered protein homeostatic signalling and atrophy in muscle (Garcia‐Martinez *et al*. 1993; Haddad *et al*. 2005). For example, inhibition of TNFα was shown to attenuate skeletal muscle proteolysis during sepsis in rodents (Zamir *et al*. 1992; Combaret *et al*. 2002). Moreover, TNFα infusion was shown to elicit MAFbx and MuRF1 upregulation in rodents (Frost *et al*. 2007). Administration of both IL‐6 and IL‐1 to rats results in increased myofibrillar protein breakdown (Zamir *et al*. 1991; Goodman, 1994). Sepsis in particular is a complex and potentially fatal condition that results from an uncontrolled, systemic inflammatory response to an infection, and is a major cause of morbidity and mortality in intensive care units (ICUs) worldwide (reviewed in Bone *et al*. 1992). Muscle wasting occurs rapidly (Puthucheary *et al*. 2013), the magnitude of which is an independent predictor of mortality in critically ill patients (Ali *et al*. 2008). Given that muscle wasting is the primary driver of subsequent physical disability in critical illness survivors (Herridge *et al*. 2003), that continues after discharge (Pfoh *et al*. 2016) with an associated mortality (Dinglas *et al*. 2017), it is appropriate that National Institute for Health and Clinical Excellence guidelines highlight physical disability and specifically its driver (muscle wasting) as a public health issue (NICE, 1990). A hallmark for the pathophysiology of sepsis is a large, uncontrolled systemic inflammatory host response to circulating microbial antigens (reviewed in Cohen, 2002), with the most common sites of infection being the lungs, urinary tract and abdominal cavity (Angus *et al*. 2001). Sepsis‐induced muscle wasting and weakness can clearly have a damaging impact on recovery and survival.

Of further interest, CRP is an acute‐phase protein of hepatic origin that increases following IL‐6 secretion by macrophages and T cells. Its physiological role is to bind to dead or dying cells (and some types of bacteria) in order to activate the complement system, and is therefore a useful marker of systemic inflammation. Circulating CRP concentration can be associated independently with loss of lean body mass (Schaap *et al*. 2006; Dutra *et al*. 2017) and age related loss of muscle mass (Schaap *et al*. 2006). Furthermore, in large observational studies CRP was not associated with age *per se*, suggesting a separate influence on muscle mass (Puzianowska‐Kuźnicka *et al*. 2016). In differentiated human myotubes, CRP has been shown to negatively affect cell size directly by decreasing muscle protein synthesis and phosphorylation of Akt and ribosomal protein S6 (Puzianowska‐Kuźnicka *et al*. 2016).

### Cellular and molecular mechanisms allied to inflammation induced muscle mass loss

A limited number of mechanistic experimental research studies focused on muscle mass loss in the context of inflammation has been conducted in patient volunteers, not least because baseline data are often difficult to acquire. This practical problem has been overcome by researchers undertaking relatively short duration endotoxin administration studies in healthy volunteers, which have reported acute decreases in both muscle protein synthesis and muscle protein breakdown, and efflux of muscle amino acids, such that muscle protein balance was unaffected (Vesali *et al*. 2009). How these observations relate to the accepted accelerated loss of muscle mass in patients under inflammatory conditions (Debigare *et al*. 2001; Puthucheary *et al*. 2013) is difficult to rationalise. One possibility is that these acute studies were performed in the post‐absorptive state when muscle protein synthesis will be reduced and muscle protein breakdown increased. However, the limited data available concerning the impact of endotoxin infusion on muscle protein balance in the post‐prandial state in humans is equally baffling as it suggests that whilst amino acid administration can rescue muscle protein loss compared to the post‐absorptive state, calculated muscle protein synthesis and breakdown rates did not differ significantly between interventions (Rittig *et al*. 2016). Clearly further research is necessary, including muscle tracer incorporation studies. In critically ill patients where stable isotope tracers were used to quantify temporal changes in muscle protein synthesis and leg protein breakdown 24 h after admission to an ICU (Puthucheary *et al*. 2013), muscle protein synthesis was found to be depressed in patients on day 1 compared with healthy control volunteers in the post‐absorptive state, but by day 7 had increased independent of nutritional status to rates similar to the post‐prandial state in control volunteers. Leg protein breakdown remained elevated throughout the study in the patients. In keeping with these observations, the authors demonstrated that muscle wasting (ultrasound determined muscle cross‐sectional area and the muscle protein‐to‐DNA ratio) occurred early and rapidly during the first week of critical illness, and was more severe among patients with multi‐organ failure compared with single organ failure. These observations of negative muscle protein balance and atrophy occurring early following ICU admittance are in keeping with data depicting elevation of muscle cytokine mRNA and widespread dephosphorylation (inactivation) of proteins regulating translation initiation factor activation and protein synthesis (Akt1, GSK3α and β, mTOR, p70S6K and 4E‐BP1) in patients within 6–8 h of admission to the ICU compared with healthy age‐ and sex‐matched control volunteers (Constantin *et al*. 2011). In accordance with the observation of elevated leg protein breakdown in such conditions (Puthucheary *et al*. 2013), this suppression of the Akt/mTOR/p70S6K signalling cascade occurred in tandem with increased muscle‐specific E3‐ligase (MAFbx and MuRF1) and 20S proteasome mRNA and protein expression levels in patients relative to controls (Constantin *et al*. 2011). The Akt/FOXO signalling pathway has been implicated in the development of muscle atrophy during catabolic conditions, potentially through activation of the ubiquitin/proteasome pathway (Crossland *et al*. 2008). More specifically, the upregulation of MAFbx and MuRF1 mRNA expression in skeletal muscle in animal models occurs within hours after the onset of endotoxaemia (Dehoux *et al*. 2003; Wray *et al*. 2003), and appears to be mediated by dampened Akt phosphorylation activating FOXO transcription factors and increased expression of downstream FOXO gene targets, including MAFbx and MuRF1 (Crossland *et al*. 2008). This series of events supports the concept that the ubiquitin/proteasome system is an important early driver of muscle protein breakdown, although not the only catabolic driver under such conditions (Deval *et al*. 2001; Smith *et al*. 2008).

Overall it would appear there are comprehensive acute alterations in muscle cross‐sectional area, muscle protein turnover and the molecular events considered to regulate muscle protein synthesis and breakdown in patients early following admittance to ICU. Although muscle mass loss is common to both immobilisation and the ICU, we do not know the relative contribution of immobilisation and inflammation to muscle mass loss in this situation. It is clear, however, that the drivers of muscle mass loss are very different between the two scenarios in humans. Furthermore, although pro‐inflammatory cytokines appear to be important in the aetiology of muscle mass loss, it is important to recognise that multiple mediators and pathways contribute to the heightened morbidity and mortality associated with infection and inflammation (reviewed in Jacobi, 2002). Indeed, anti‐cytokine therapies were developed to dampen the systemic inflammatory response, including antibodies against TNFα, soluble TNFα receptors and IL‐1β receptor antagonists. Blockade of TNFα was found to have beneficial effects on survival in animal models of shock (Beutler *et al*. 1985; Tracey *et al*. 1987); however, subsequent clinical trials did not show benefit in human sepsis (Reinhart & Karzai, 2001). Furthermore, IL‐1β inhibition using a recombinant receptor antagonist reduced mortality in animal models of shock (Ohlsson *et al*. 1990), but, again, human trials showed no beneficial effects (Opal *et al*. 1997).

### Inflammation induced muscle insulin resistance

Various catabolic conditions that stimulate muscle atrophy are often associated with insulin resistance, including heightened muscle inflammation (Hasselgren *et al*. 1987; Dardevet *et al*. 1994; Wang *et al*. 2006). Insulin‐stimulated whole body glucose disposal, endogenous glucose production and glucose oxidation have been shown to be impaired in patients with severe sepsis (Chambrier *et al*. 2000), which is associated with increased mortality (Falciglia *et al*. 2009).

### Cellular and molecular mechanisms associated with inflammation induced muscle insulin resistance

Although current practice is to maintain normoglycaemia in intensive care, detailed mechanistic insight regarding the aetiology of this patient phenotype is missing and current insight has been gained in the main from animal based investigation. Hyperlactataemia is also a frequent manifestation of sepsis, which, based on preclinical research, may be at least partially due to impaired PDC activity and flux limiting mitochondrial pyruvate utilisation as a result of sepsis induced PDK upregulation (Vary, 1999; Alamdari *et al*. 2008). Given the proposed role of TNFα in insulin resistance (Plomgaard *et al*. 2005), possibly through suppression of insulin signalling at the level of IRS‐1 (Hotamisligil *et al*. 1996; del Aguila *et al*. 1999), it is more than likely that elevated cytokines have some role in the observed changes in carbohydrate metabolism in inflammatory states, and at least partly through dysregulation of Akt signalling (Crossland *et al*. 2008), not least because divergent *in vivo* pharmacological approaches that reduce muscle inflammation blunt the dysregulation of muscle carbohydrate metabolism (Crossland *et al*. 2010, 2017).

It is also of note that a link between circulating CRP and insulin resistance has been established, with associations seen with measures of fat mass, fasting insulin and a number of metabolic disorders (Festa *et al*. 2000). Just as with protein homeostasis, circulating CRP may not be simply a biomarker of inflammation but may play a causal role in the dysregulation of fuel metabolism. CRP binds and aggregates low density lipoproteins and very low density lipoproteins (de Beer *et al*. 1982). Additionally, CRP impacts on macrophage phenotype (Dong & Wright, 1996), which purportedly impacts on insulin resistance. However, a direct link between CRP and insulin resistance remains elusive, and causal links have not been established with type 2 diabetes (Brunner *et al*. 2008). CRP is regulated by other pro‐inflammatory cytokines such as TNFα and IL‐6 for which more robust direct causative links have been established (see above), and as such circulating CRP may be an upstream representative of their activity (Ridker, 2016).

### Molecular cross‐talk between muscle atrophy and insulin signalling in inflammation

Few studies to date have investigated muscle protein and fuel metabolism concurrently to elucidate potential mutual signalling events that may explain dysregulation of muscle protein and carbohydrate metabolism in inflammation. As previously discussed, Akt signalling appears to be crucial in hypertrophy and atrophy signalling, as well as insulin signalling, and it is increasingly evident that there is cross‐talk between insulin and atrophy signalling processes during heightened inflammation in animal models (Crossland *et al*. 2008) and patients (Constantin *et al*. 2011). Specifically, elevated cytokines may result in the impairment (reduced phosphorylation) of Akt1 via inhibition of IRS‐1 (del Aguila *et al*. 1999), subsequently leading to the dephosphorylation (activation) of FOXO, and, in turn, transcriptional upregulation of FOXO target genes MAFbx, MuRF1 and PDK, as well as decreased phosphorylation (activation) of muscle anabolic signalling proteins (Crossland *et al*. 2008; Constantin *et al*. 2011). In support of this, genetically insulin‐resistant, *db/db*, mice were found to have increased rates of muscle proteolysis, which correlated with altered Akt/FOXO signalling (Wang *et al*. 2006). Furthermore, strategies aimed at blunting the muscle cytokine response to inflammation *in vivo* in an animal model of clinical sepsis have been shown to dampen the dysregulation of Akt/FOXO signalling and the abundance of downstream mRNA targets, whilst concomitantly preventing muscle protein loss and the impairment of pyruvate dehydrogenase complex activation and carbohydrate oxidation (Crossland *et al*. 2010, 2017). Collectively, these findings, along with other reports of dysregulation of Akt signalling in the catabolic, insulin resistant state (Wang *et al*. 2006), and the proposed role of FOXO in both atrophy and insulin signalling pathways (Kwon *et al*. 2004; Sandri *et al*. 2004), suggest that Akt and FOXO form part of a common signalling pathway influencing muscle protein breakdown, protein synthesis and the induction of insulin resistance during inflammation (Abstract figure).

## Conclusion

This review highlights that whilst muscle mass loss and insulin resistance are common end‐points of immobilisation and increased inflammatory burden, there is a lack of understanding of the mechanisms responsible for these common events such that a substantial gap in understanding of the pathophysiology exists. It does seem unlikely, however, that the same mechanisms are involved. For example, Akt signalling appears to play a central role in the dysregulation of protein metabolism in conditions of increased inflammatory burden, but not in immobilisation. It is important therefore for future research to determine the mechanisms responsible for the loss of muscle mass and insulin sensitivity during immobilisation and whether the impacts of combined immobilisation and increased inflammatory burden are additive in situations such as intensive care, which could have important clinical ramifications. Importantly, these studies need to be conducted in humans, and will include multiple time‐point measurements over the course of intervention, dynamic measurements of muscle protein turnover and glucose uptake in the post‐prandial state, and will be combined with sensitive measures of muscle composition, intermediary metabolism and modern molecular biology. Such studies will produce major new mechanistic insights into human pathophysiology, with potential application to new therapeutic approaches and opportunities for “reverse translation” to more basic research.

## Additional information

### Competing interests

None declared.

### Author contributions

P.L.G. wrote the first draft of the review manuscript, which all other authors added to and edited. D.C.‐T. created the Abstract figure, which P.L.G. edited. All authors approved the final version of the article. All persons designated as authors qualify for authorship, and all those who are eligible for authorship are listed.

### Funding

This work was supported by the Medical Research Council [grant number MR/K00414X/1]; and Arthritis Research UK [grant number 19891, 21595] Centre for Musculoskeletal Ageing Research, the Arthritis Research UK Centre for Sport, Exercise and Osteoarthritis, the Biotechnology and Biological Sciences Research Council and the National Institute for Health Research Nottingham Biomedical Research Centre by contributions to the infrastructure that facilitated generation of this review article.
